# A Slot Blot Immunoassay for Quantitative Detection of *Plasmodium falciparum* Circumsporozoite Protein in Mosquito Midgut Oocyst

**DOI:** 10.1371/journal.pone.0115807

**Published:** 2014-12-22

**Authors:** Sanjai Kumar, Hong Zheng, Bingbing Deng, Babita Mahajan, Bryan Grabias, Yukiko Kozakai, Merribeth J. Morin, Emily Locke, Ashley Birkett, Kazutoyo Miura, Carole Long

**Affiliations:** 1 Laboratory of Emerging Pathogens, Division of Emerging and Transfusion Transmitted Diseases, Center for Biologics Evaluation and Research, Food and Drug Administration, Silver Spring, Maryland 20993, United States of America; 2 Laboratory of Malaria and Vector Research, National Institute of Allergy and Infectious Diseases, National Institutes of Health, Rockville, Maryland 20852, United States of America; 3 Program for Appropriate Technology in Health (PATH) Malaria Vaccine Initiative, Washington, DC 20001, United States of America; Johns Hopkins University, United States of America

## Abstract

There is still a need for sensitive and reproducible immunoassays for quantitative detection of malarial antigens in preclinical and clinical phases of vaccine development and in epidemiology and surveillance studies, particularly in the vector host. Here we report the results of sensitivity and reproducibility studies for a research-grade, quantitative enhanced chemiluminescent-based slot blot assay (ECL-SB) for detection of both recombinant *Plasmodium falciparum* circumsporozoite protein (r*Pf*CSP) and native *Pf*CSP from Oocysts (*Pf* Oocyst) developing in the midguts of *Anopheles stephensi* mosquitoes. The ECL-SB detects as little as 1.25 pg of r*Pf*CSP (linear range of quantitation 2.5–20 pg; R^2^ = 0.9505). We also find the earliest detectable expression of native *Pf*CSP in *Pf* Oocyst by ECL-SB occurs on day 7 post feeding with infected blood meal. The ECL-SB was able to detect approximately as few as 0.5 day 8 *Pf* Oocyst*s* (linear quantitation range 1–4, R^2^ = 0.9795) and determined that one *Pf* Oocyst expressed approximately 2.0 pg (0.5–3 pg) of native *Pf*CSP, suggesting a similar range of detection for recombinant and native forms of *Pf* CSP. The ECL-SB is highly reproducible; the Coefficient of Variation (CV) for inter-assay variability for r*Pf* CSP and native *Pf*CSP were 1.74% and 1.32%, respectively. The CVs for intra-assay variability performed on three days for r*Pf* CSP were 2.41%, 0.82% and 2% and for native *Pf* CSP 1.52%, 0.57%, and 1.86%, respectively. In addition, the ECL-SB was comparable to microscopy in determining the *P. falciparum* prevalence in mosquito populations that distinctly contained either high and low midgut *Pf* Oocyst burden. In whole mosquito samples, estimations of positivity for *P. falciparum* in the high and low burden groups were 83.3% and 23.3% by ECL-SB and 85.7% and 27.6% by microscopy. Based on its performance characteristics, ECL-SB could be valuable in vaccine development and to measure the parasite prevalence in mosquitoes and transmission-blocking interventions in endemic areas.

## Introduction

Highly sensitive and reproducible immunoassays that are amenable to high throughput adaptation are a critical need for diagnostics, vaccine development and manufacturing, and in pathogen surveillance and epidemiology studies. Some of the prerequisites for such assays to meet the needs of these applications include 1) a non-variant antigen/epitope for detection; 2) a quantitative or semi-quantitative nature; and 3) ease of operation, data recording and analysis.

In malaria, vaccine development efforts are hampered due to the scarcity of standardized assays to support the process from antigen discovery to clinical development of candidate vaccines. These challenges are more acute for mosquito stages of parasite development. During this phase, protective antibodies are thought to disrupt parasite development by preventing the male and female gametes from entering into fertilization, interfering with zygote formation or further transition from the ookinete to midgut oocyst stages where primordial sporozoites are formed and undergo maturation before their migration to salivary glands. Both naturally acquired [1]–[3] and vaccination- induced antibodies [4]–[7] can interrupt parasite development in mosquitoes resulting in reduced or blocked parasite transmission. Currently the efficacy of transmission-blocking antibodies is measured in an *ex vivo* assay, the standard membrane feeding assay (SMFA), based on enumerating the number of oocysts that develop inside the midgut of mosquitoes with prevention or reduction in oocyst intensity as the readout [8], [9]. This assay is considered to be biologically relevant because it allows measurement of the transmission reducing activity of antibodies taken up by the mosquito during feeding. Currently, measurement of transmission-reducing activity requires dissection of the mosquito midgut to visualize and enumerate oocysts by microscopy. This is a highly labor-intensive, cumbersome, and possibly error prone process [9], and thus has severe limitations and cannot be applied in a high throughput manner especially in large clinical studies involving hundreds or thousands of volunteers. Therefore, sensitive immunological assays not based on microscopy are needed to assess the impact of vaccine and drug interventions on the intensity and prevalence of *Plasmodium* infection in mosquitoes as well as for epidemiological studies.

In the recent years, efforts have been made to develop assays to measure and quantify *Plasmodium* infection rates in mosquitoes in the laboratory and field settings in high throughput formats. One report has utilized a transgenic luciferase-expressing *P. falciparum* strain for high throughput measurement of oocyst burden in blood meal fed mosquitoes in the laboratory setting [10]. Another method that may be amiable to high throughput adaptation applies the PCR technology for the 18S rRNA-based quantification of *Plasmodium* parasites developing in the mosquito midguts [11]. Several enzyme-linked immunosorbent assays (ELISAs) based on detection of circumsporozoite protein (CSP) have also been reported that detect *Plasmodium* sporozoites in anopheline mosquitoes [12]–[14]. However, the analytical sensitivity and ability to detect developing midgut oocysts by these ELISA tests has never been established. Moreover, high false positive CSP-ELISA results have been reported for *P. falciparum* and *P. vivax* sporozoites [15], particularly when testing for *P. falciparum* in vectors that have zoophilic biting trends [12], [16].

Recently, we have reported an enhanced chemiluminescent Western Blot (ECL-WB) that detected *P. falciparum* CSP (*Pf* CSP) in the range of 3–12 pg of protein, with inter-assay variability Coefficient of Variation (CV%) of 10.31% and mean intra-assay CV% of 3.16% [17]. In this communication, we report the development of a highly sensitive enhanced chemiluminescent slot blot (ECL-SB) based on quantitative detection of *Pf* CSP using an anti-*Pf* CSP mAb 2A10 [18] that recognizes the CSP repeat NANP unit. This antigen-antibody reaction was visualized by incubation with a chemiluminescent detection system and band intensity was measured as integrated optical density (IOD). A standard curve from IOD values obtained from known concentrations of *Pf* CSP was generated which was then used to convert the IOD values from test samples into quantitative measurement of *Pf* CSP. Assay sensitivity and reproducibility was established using *E. coli* expressed recombinant *Pf* CSP and *A. stephensi* midguts isolated on day 8 post-*P. falciparum* infected blood meal. The choice of *Pf* CSP as detection antigen was based on its expression in oocysts from day 7 onwards post-blood meal through mature sporozoites. The conserved nature of NANP repeat sequence in all *P. falciparum* isolates sequenced to date [19], [20] make this a suitable target for detecting parasites from different geographical regions of the world. Thus, a CSP-NANP based ECL-SB should be applicable to detection of all *P. falciparum* strains developing in the midguts of *Anopheles* mosquitoes independent of the vector's species or genotype.

We find that the ECL-SB is comparable to the ECL-WB in sensitivity and reproducibility, but significantly superior in terms of ease of operation and potential for high throughput adaptation. Thus, this assay may be used as a replacement/supplement for a Western blot, or other similar immunological assays in preclinical and clinical vaccine development processes for applications such as antigen discovery, assessment of immune responses or quantification of antigenic component(s) in a vaccine formulation.

## Materials and Methods

### r*Pf* CSP

Recombinant *P. falciparum* CSP (r*Pf*CSP) amino acid sequence 27-123[NANPNVDP]_3_[NANP]_21_300-411 expressed in *E. coli* and purified on a heparin sepharose affinity column was used as source of recombinant antigen. The details for the recombinant expression, purification and antigenic characterization of r*Pf* CSP are described earlier [17], [21]. Protein estimation of r*Pf*CSP was performed by using the Coomassie Protein Assay Reagent (Thomas Scientific, 1856209, Swedesboro, NJ). The protein concentration of the batch of r*Pf* CSP used in this study was 550 µg/ml and further diluted to create a stock concentration of 1ng/100 µl in 1X SDS-PAGE loading dye and then two-fold serial dilutions were prepared from these stock samples and stored at -80°C until further use.

### mAb 2A10

A large batch of anti-*Pf* CSP mAb 2A10 was generated from a hybridoma cell line acquired from the MR4/ATCC, Virginia [18]. Ascites production in mice and antibody purification using Protein G affinity chromatography was accomplished by using a commercial source (Harlan Laboratories Inc. Madison, WI). mAb 2A10 (1.55 mg/ml) was characterized for its immune-reactivity in IFA using *P. falciparum* sporozoites and in ELISA and Western Blot using r*Pf* CSP.

### Production and Enumeration of *P. falciparum* Oocysts

Three to five day old female *A. stephensi* (Nijmegen strain) mosquitoes were membrane fed with *P. falciparum* (NF54) gametocytes that were prepared by enrichment of asexual blood stage *P. falciparum* parasites cultured in human erythrocytes and serum [8]. The human erythrocytes and plasma used for the *P. falciparum* cultures were purchased from Interstate Blood Bank, Inc. (Memphis, TN: www.interstatebloodbank.com). The cages containing gametocyte fed mosquitoes were maintained at 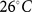, 80% relative humidity and were kept for up to nine days after feeding to allow parasites to develop into mature oocysts. Infectivity was measured seven to nine days after blood meal, by randomly sampling 20 mosquitoes and harvesting the midguts. Only midguts from mosquitoes with eggs at the time of dissection were analyzed. Midguts were stained with 0.05% mercurochrome solution for 20-45 minutes on a regular microscope slide in a drop of mercurochrome and the number of *Pf* Oocysts per midgut was counted by two independent examiners. An equal number of midguts were also harvested from the same lot of mosquitoes that had received a blood meal containing *P. falciparum* gametocytes but without mercurochrome staining and were assigned a *Pf* Oocyst score based on the arithmetic mean of *Pf* Oocysts determined by mercurochrome staining.

### *Pf* CSP Detection in *Pf* Oocyst

*A. stephensi* midguts containing *Pf* Oocysts, scored by microscopy or un-scored, were washed with 1X PBS three times. The midgut samples were boiled at 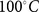 for five minutes, lysate was centrifuged at 13,000 rpm for two minutes, and supernatant was collected. A stock solution of eight *Pf* Oocysts/20 µl was prepared in TBS-0.5% SDS buffer utilizing mosquito midguts with estimated oocyst intensities and further two-fold serial dilutions of the lysates to achieve the equivalent of 4 to 0.125 *Pf* Oocyst/20 µl TBS-SDS buffer was prepared for use in ECL-SB.

### *Pf* CSP Detection in Whole Mosquito Lysates

We developed a method to use the whole mosquito sample for *Pf* CSP detection by ECL-SB. To accomplish this, mature *in vitro* cultured *P. falciparum* gametocytes (NF54 isolate) were fed through a membrane-feeding apparatus to two independent populations of *A. stephensi* (Nijmegen strain) mosquitoes. The two populations differed as they were fed with the blood meals containing the high (0.035%) or low (0.009%) counts of gametocytes to create the high and low infectivity rate in the mosquito midguts. Mosquito samples were harvested on 8 days post blood meal for both microscopic dissection and slot blot analysis. The determination of *Pf* Oocyst prevalence in the two sets of mosquito populations were determined by microscopic counting of stained midguts as described above. For the estimation of *P. falciparum* prevalence by ECL-SB, *Pf* CSP expression on the developing oocysts was determined on lysed whole mosquitoes. Briefly, each individual uninfected or infected mosquito was placed into a separate tube and homogenized with a piston in 50 µL of lysis buffer (1X TBS, 0.5% SDS). The lysates were subsequently vortexed for 20 s and then boiled for 5 minutes. Any particulate matter was pelleted via centrifugation and the supernatant was collected and analyzed.

### ECL-Western Blot (ECL-WB)

This assay was performed by using the procedure described earlier by our laboratory [17]. r*Pf* CSP and midgut lysates were used for antigen detection. mAb 2A10 (at 0.31 µg/ml) was used as primary antibody and an ECL-goat anti-mouse-IgM+IgG conjugated to AP (1∶5000 dilution); as source of secondary antibody. Finally, the membrane was incubated with the ECL substrate solution (Life Technologies,Western-Star^TM^ Immunodetection System, T1046, Grand Island, NY) and exposed to an autoradiograph (AR) film (KODAK X-OMAT AR, IB1651579; Radnor, PA) and developed using a Kodak developer (X-OMAT 1000A).

### ECL-Slot Blot

This assay was performed using a Minifold 48 slots, Whatman apparatus (GE Healthcare Life Sciences, 10447941; Piscataway, NJ). Two-fold serial dilutions of r*Pf* CSP (ranging from 40–1.25 pg) and *Pf* Oocyst lysate (ranging from 4–0.25 *Pf* Oocyst*s*/20 µl) in TBS plus 0.5% SDS were loaded in duplicate in the wells in the slot blot apparatus. The protein samples were allowed to bind to nitrocellulose paper for one hour and then slots were washed with 500 µl of PBS four times. The membrane was blocked at RT in iBlock blocking buffer (Applied Biosystems, T2015, Foster City, CA) for one hour and probed with anti-*Pf* CSP mAb 2A10 (at 0.31 µg/ml) followed by three washings (five minutes each) in PBS and three washings in iBlock blocking buffer. Next, the membrane was incubated with an ECL-goat anti-mouse- IgM+IgG conjugated to AP (1∶5000 dilution) for one hour at RT and then washed again as described following incubation with mAb 2A10. Next, the membrane was rinsed twice for two minutes with 25 ml of 1X assay buffer and then incubated with 6 ml of ECL-substrate solution at RT for five minutes and bands were visualized by exposure to an AR film and developed (Kodak X-OMAT 1000A). The ECL-reagents used in this assay were purchased as a kit (Life Technologies, Western-StarTM Immunodetection System, T1046, Grand Island, NY).

**Figure 1:**
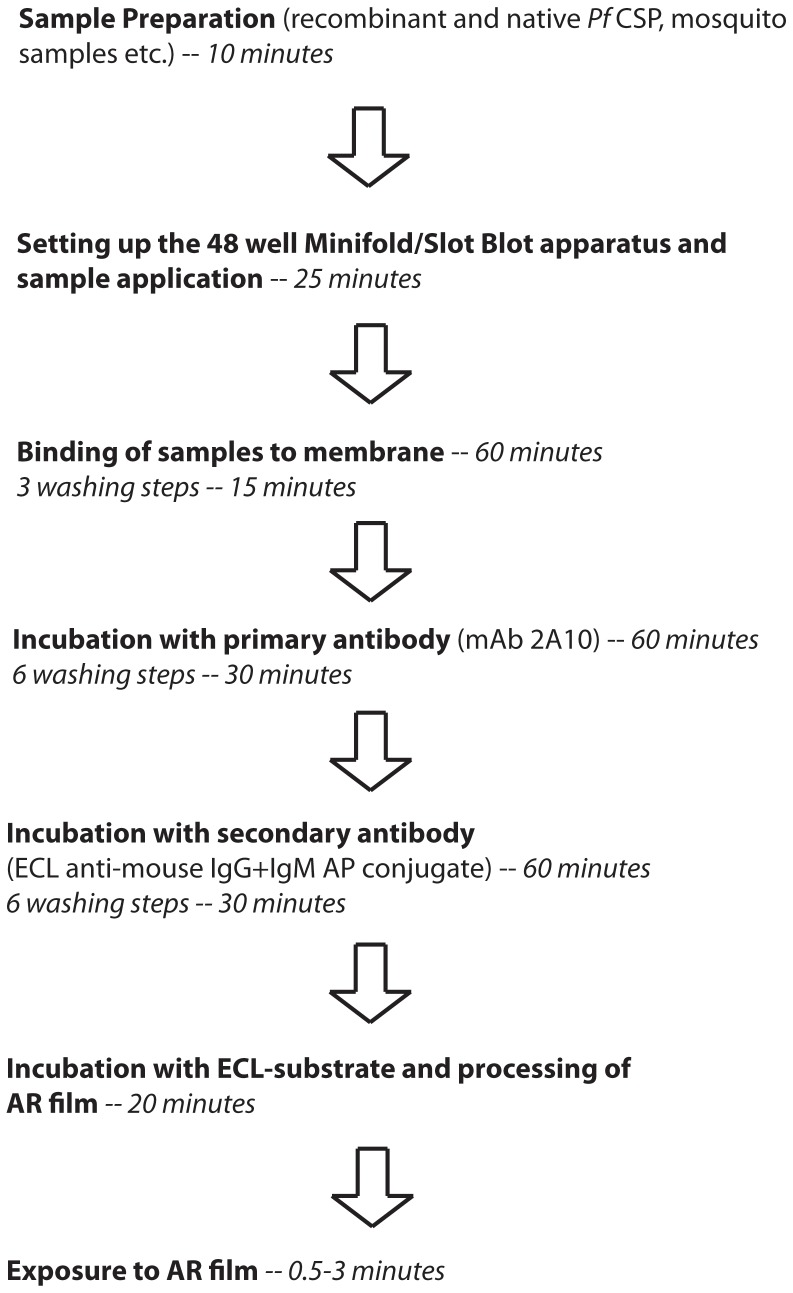
Outline of the Enhanced Chemiluminescent-Slot Blot (ECL-SB) experimental procedure.

A brief step-wise description of the procedure to perform the slot blot assay and the time taken to complete each step is shown in Fig. 1.

### Data Acquisition and Analysis

The band profile on the film was scanned and analyzed using the ImageJ program (http://rsbweb.nih.gov/ij/). The integrated optical density (IOD) of each band was determined by measuring the band intensity in a ‘gated area’. The dimensions of the gated area for IOD determination was kept constant for each band on the scanned image.

## Statistical Analysis

### Detection of *Pf* CSP

The Michaelis-Menten (M-M) equation [22] was applied to estimate the IOD corresponding to the concentration of r*Pf* CSP using ECL-SB. The M-M equation is a non-linear model which indicated that the IOD approached a saturation level as the amount of r*Pf* CSP reached a specific concentration. This equation is defined as 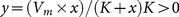
(1)where 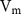 is the horizontal asymptote of IOD and 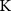 is the concentration of r*Pf*CSP (pg) at which the IOD is a half of the horizontal asymptote. The M-M equation was used to define the relationship between IOD (y) and r*Pf* CSP (x) and between IOD and amount of *Pf* CSP on *Pf* Oocyst.

### Precision

We measured the intra-assay variability (within run) and the inter-assay variability (between runs) as two distinct measurements to determine the ability of the ECL-SB to consistently detect *E. coli* expressed r*Pf* CSP and native *Pf* CSP on *Pf* Oocyst. To determine the intra-assay variability, 5 pg of r*Pf*CSP or 4 *Pf* Oocyst was run eight times in each experiment. The mean and the standard deviation (SD) of IODs obtained from those eight runs were used for the calculation. The inter-assay variability was calculated by using the means of eight runs obtained on three different days. The assay precision was calculated as percent of CV. 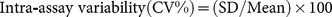
where the mean and standard deviation (SD) were calculated among samples in the same experiment, and
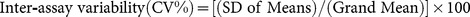
where the SD of Means and Grand Mean was calculated in experiments conducted on three days.

### Comparison of *Pf* CSP Expression in Oocysts Using Stained and Unstained Midgut Samples

The median *Pf* CSP expression on stained and unstained infected midguts (day 8 post-blood-meal) was compared in a total of 18 samples from each test group using the Mann-Whitney test.

### Comparison Between Microscopy and ECL-SB for Prevalence Estimation in Vector Populations

The strength of the correlation between the overall diagnostic results of these two distinct methods of analysis was confirmed using a chi squared test for homogeneity.

## Results and Discussion

### Dynamics of *Pf* CSP Expression on *Pf* Oocysts During the Developmental Stages in the Mosquito Midgut

**Figure 2:**
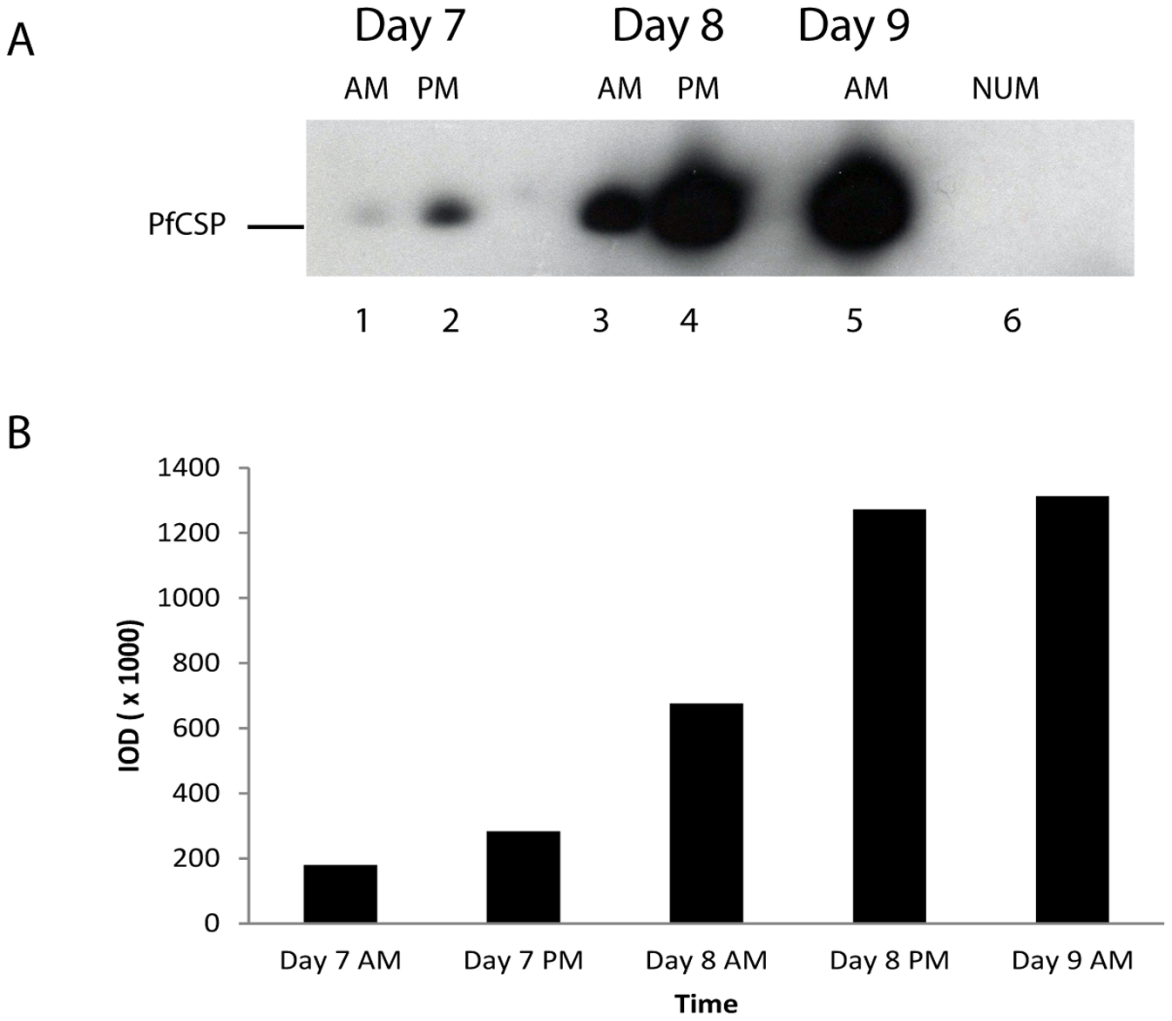
The dynamics of circumsporozoite protein (CSP) expression on developing *P. falciparum* oocysts in the mosquito midgut. A: Midguts from 10 infected or uninfected mosquitoes were dissected on days 7–9 post feeding. Lysates were prepared by boiling the midguts in 1X SDS-PAGE gel loading dye for 5 minutes (2 midguts/10 µl dye). 10 µl of the sample was subjected to electrophoresis on 4–12% NuPAGE gel and transferred onto a PVDF membrane and probed with the mAb 2A10. No midgut was collected on the evening of day 9 due to saturation of *Pf* CSP expression levels. Lanes 1–5: *Pf* Oocysts from infected mosquitoes; Lane 6: Lysate from normal uninfected midgut (NUM). AM – Midgut samples collected around 10.00 AM; PM – Midgut samples collected around 3.00 PM. B: The amount of protein was determined by measuring the intensity of a specific band using the ImageJ software and was represented as integrated optical density (IOD).

The development of infectious sporozoites progresses for 10 to 14 days through the vegetative oocyst stage in the *Anopheline* mosquito midgut. CSP is the most predominant parasite protein in developing and mature sporozoites [23], [24]. In order to facilitate the development of CSP-based immunoassays for parasite detection in the midgut, we wanted to know the dynamics of CSP expression during the midgut developmental stages. To accomplish this, we determined the expression of *Pf*CSP on oocysts from *A. stephensi* midguts collected twice (AM and PM) every day between days 7 to 9 post blood meal in the ECL-WB. Midguts from uninfected mosquitoes were used as controls. Midgut lysates were prepared and probed with anti-*Pf* CSP mAb 2A10 (at 0.31 µg/ml concentration). Ten midguts from each experimental group were pooled, lysed and a total amount of protein equivalent to two midguts in 10 µl of SDS- loading dye was applied into each lane of a 4–20% gradient PAG. The first detectable expression of *Pf* CSP was observed on day 7 AM (Fig. 2A), and optimal detectable label was reached on day 8 AM. The expression level substantially increased by day 8 PM and reached detection saturation on day 9 post blood meal (Fig. 2A and B). Based on the detectable expression range, the day 8 AM infected midguts were used for further studies.

### Range of Detection of r*Pf* CSP by ECL-SB

**Figure 3:**
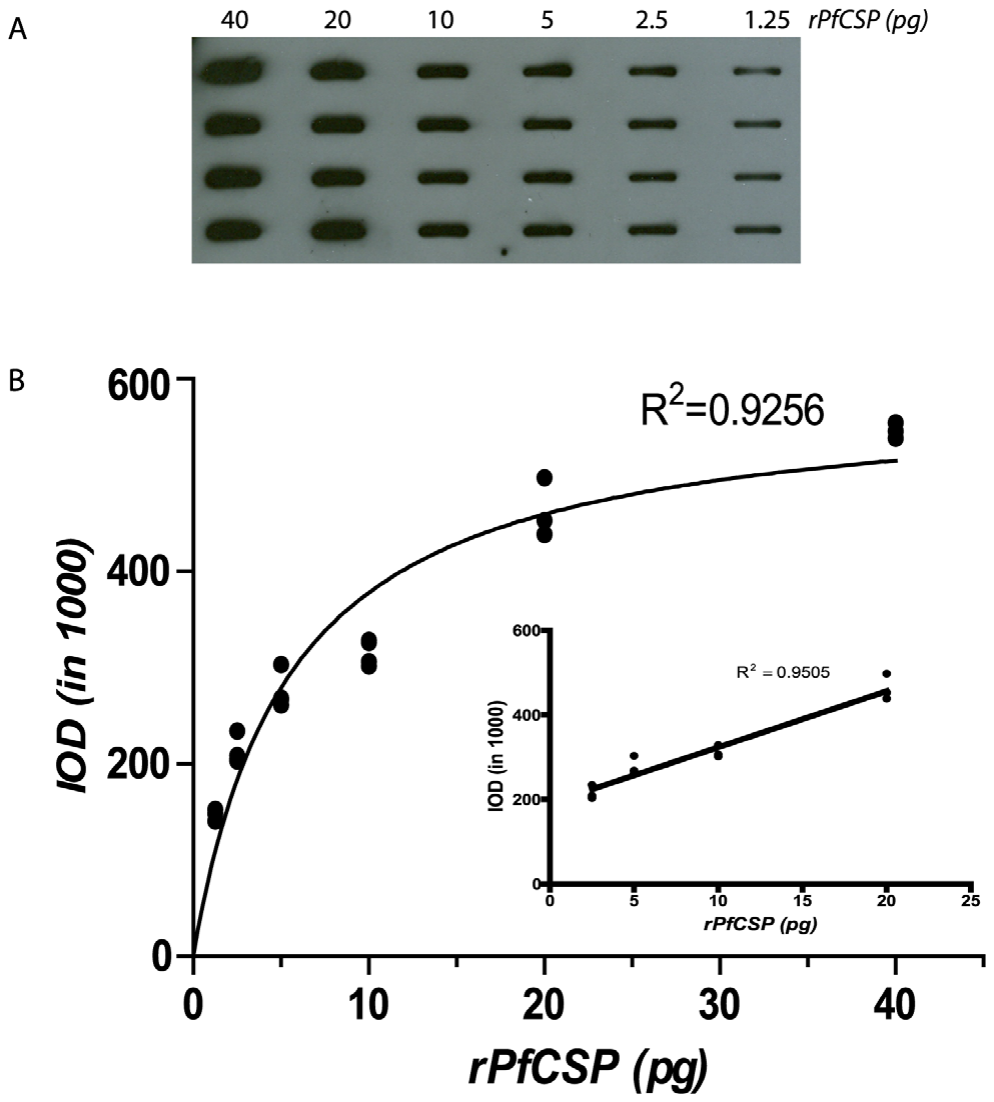
Range of detection of r*Pf*CSP in the ECL-SB. A: Two-fold dilutions of r*Pf* CSP were transferred onto a nitrocellulose membrane using a slot blot apparatus and the membrane was probed with the mAb 2A10. The amount of protein was determined by measuring the intensity of a specific band using the ImageJ software and was represented as integrated optical density (IOD). B: A standard curve based on the IOD values was derived using the Michaelis–Menten hyperbolic function. The linear range of antigen–antibody reaction shown in inset is obtained with 2.5–20 pg of r*Pf* CSP.

Initial feasibility and optimization studies for quantitative detection of a malarial antigen by ECL-SB were determined by using r*Pf* CSP. Two-fold serial dilutions of r*Pf* CSP (40 to1.25 pg) were run on the ECL-SB to determine the optimal range of quantitation. Fig. 3A shows that a discrete band is visible when as little as 1.25 pg of r*Pf*CSP is run on the ECL-SB. We fitted the IOD values obtained from six known concentrations of r*Pf* CSP (1.25, 2.5, 5, 10, 20 and 40 pg) using M-M equation (1) as shown in Fig. 3B. The association between the amount of r*Pf* CSP and IOD is measured by the coefficient of determination (R^2^), and M-M equation shows a reasonable fit (R^2^ = 0.9256). Fig. 3B shows a part of the curve where a linear relationship was found at the r*Pf* CSP concentrations between 2.5 to 20 pg and R^2^ = 0.9505. We calculated that, within in the linear range, the rate of IOD increment per pg of r*Pf* CSP was 13280 suggesting a constant trend between IOD value and r*Pf* CSP concentration. Overall, these results demonstrate that ECL-SB is capable of detecting very low amounts of r*Pf* CSP.

### Relationship between ECL-SB and ECL-WB

**Figure 4:**
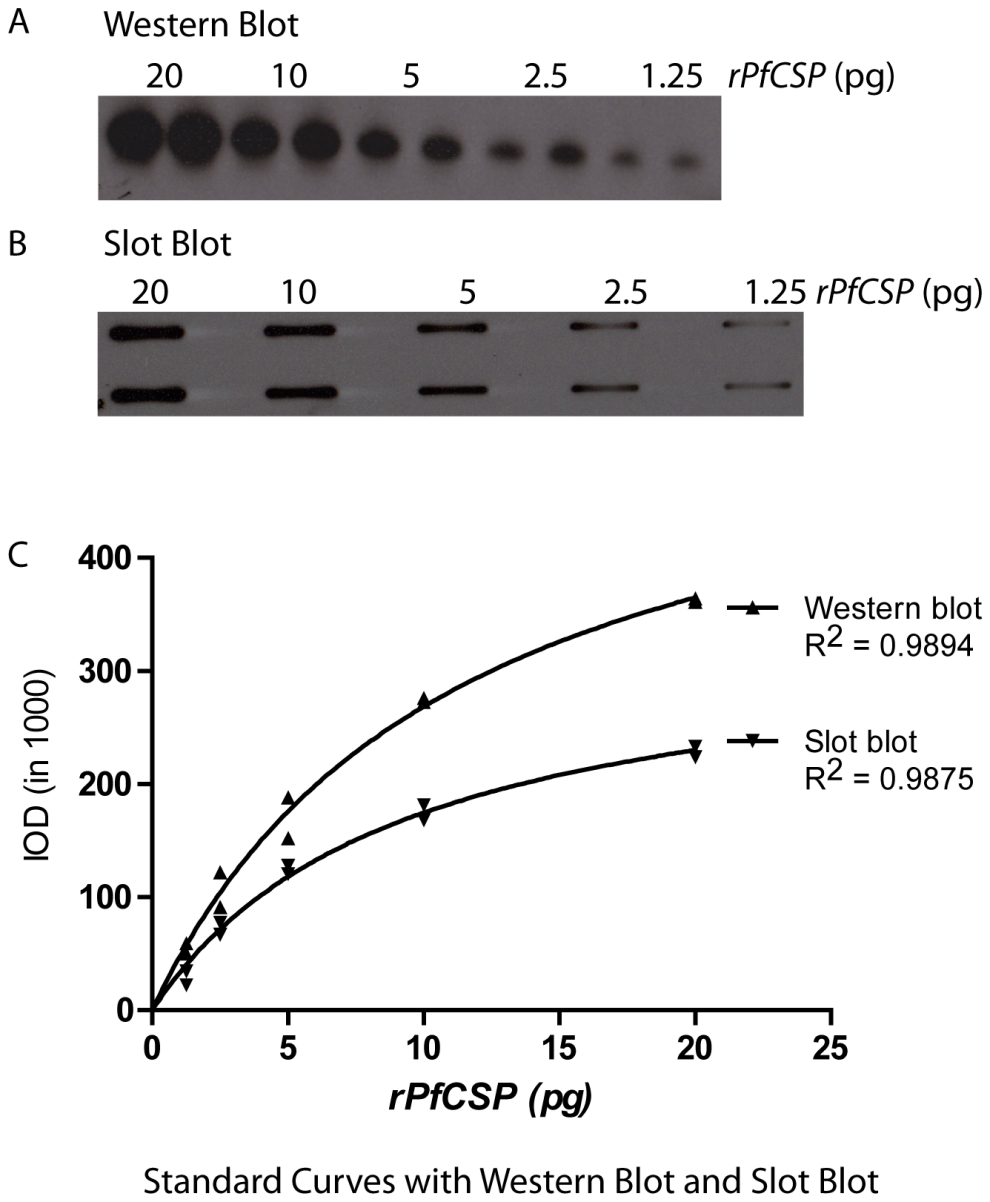
Relationship between ECL-Western blot (ECL-WB) and ECL-SB. The IOD values obtained from ECL-WB (A) and ECL-SB (B) analysis of two-fold dilutions of r*Pf* CSP (20 pg -1.25 pg) were overlaid (C).

We determined the comparative sensitivity of ECL-SB and ECL-WB for detection of r*Pf* CSP. The five known concentrations of r*Pf* CSP (1.25, 2.5, 5, 10 and 20 pg) were run in both assays and the IOD values were obtained. Both assays were able to detect as little as 1.25 pg of r*Pf* CSP (Figs. 4A and 4B). A relationship between the ECL-SB and ECL-WB was established by overlaying the M-M curves obtained from IOD values of two-fold dilutions of *Pf* CSP (Fig. 4C). Results showed that the IOD values from the slot blot increased less rapidly than the IOD values from the Western blot. In order to understand how IOD values associated with the r*Pf* CSP concentration, we fitted the predicted IOD values obtained in the two assays by using the linear model. The slope of the line indicated that the slot blot IOD values increased on average by 0.63 in relation to one IOD unit increase to Western Blot. Overall, these results suggest that both assays possess comparable sensitivity in detecting r*Pf* CSP.

### Range of Detection of *Pf* Oocyst

**Figure 5:**
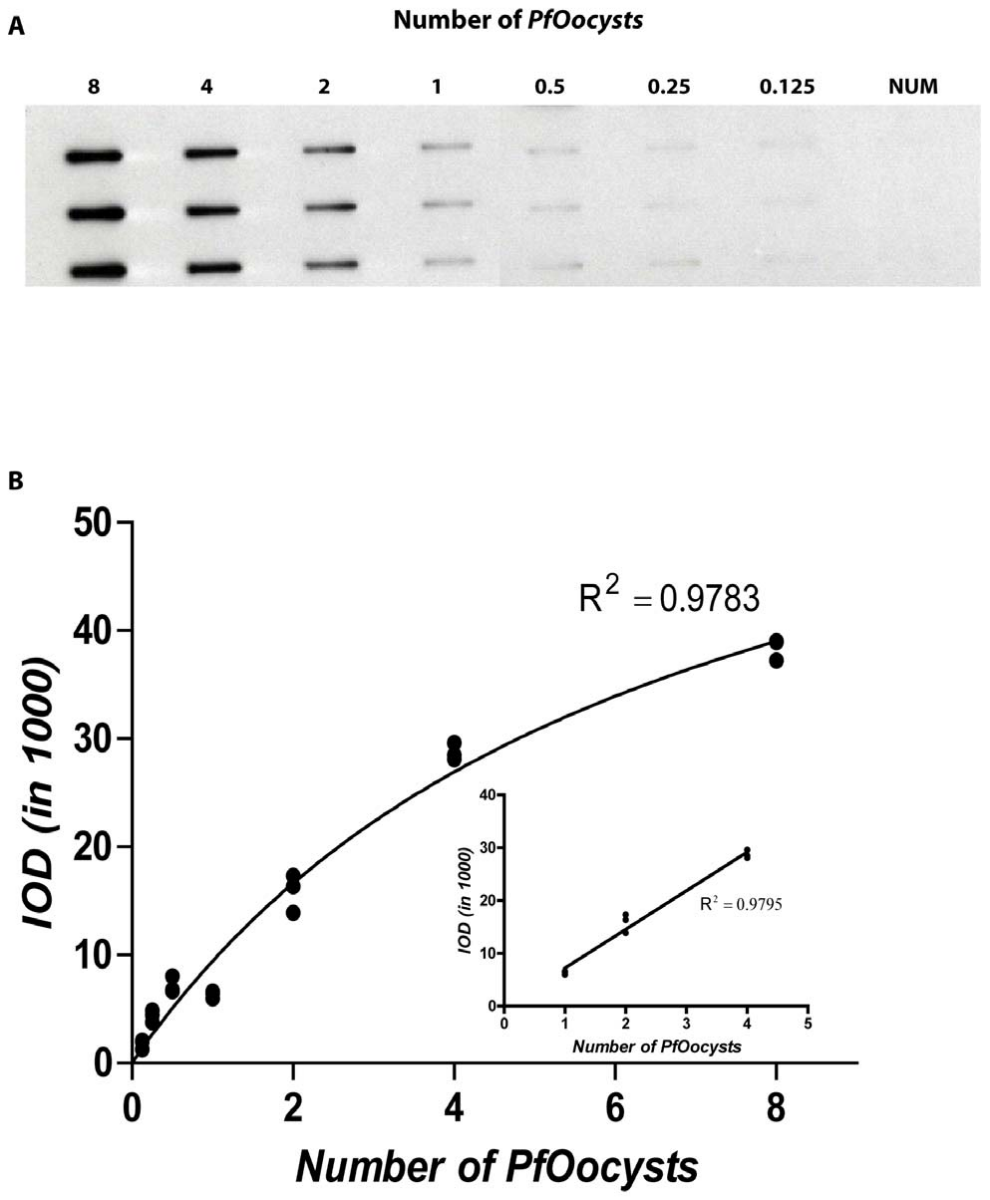
Range of detection of *Pf* Oocyst by ECL-SB. A: Two-fold dilutions of *Pf* Oocysts were transferred onto a nitrocellulose membrane using a slot blot apparatus and probed with the mAb 2A10. The amount of protein was determined by measuring the intensity of a specific band using ImageJ software and was represented as integrated optical density (IOD). B: A standard curve based on the IOD values was derived using the Michaelis–Menten hyperbolic function. The linear range of antigen–antibody reaction shown in the inset is obtained within 1–4 *Pf* Oocysts.

In the SMFA, microscopic enumeration of *Pf* Oocyst is generally performed on day 8 which coincides with the optimal *Pf* CSP expression as measured by ECL-WB on day 8. We wanted to explore the utility of *Pf* CSP expressed on day 8 *Pf* Oocysts as a marker for estimation of *Pf* Oocyst burden in the midgut. To accomplish this, we determined the range of quantitation of *Pf* Oocysts by quantitating *Pf* CSP expression in unstained, pooled midguts (see Materials and Methods). Two-fold serial dilutions of *Pf* Oocysts (8 to 0.125 *Pf* Oocysts) were loaded on the slot blot and, following reactivity with 2A10 mAb and visualization with ECL-reagents, the corresponding IOD values were determined. Fig. 5A shows very clear reactivity with as little as one *Pf* Oocyst. The IOD values were obtained from the dilution of *Pf* Oocyst counts (0.125, 0.25, 0.5, 1, 2, 4, and 8) and the M-M equation (1) was applied to determine the linear range of quantitation (R^2^ = 0.9795, Fig. 5B). While a clear, detectable band was observed with as little as 0.25 *Pf* Oocyst, the linear quantitation range was between one and four *Pf* Oocyst (Fig. 5A and B). Cumulatively, the sensitivity of the ECL-SB assay is sufficient to discriminate between the *P. falciparum* positive and P. falciparum negative mosquitoes that had received an infected blood meal. However, because the linear detection range lies between 1-4 *Pf* Oocyst, this assay may be less reliable for quantification of high oocyst burdens sometime observed in the SMFA.

### Quantitation of *Pf* CSP Expression on *Pf* Oocyst

Reduction in the number of oocysts developing in the mosquito midgut is the standard readout for measurements of the transmission reducing efficacy of candidate vaccines in the SMFA. We have shown that the optimally detectable amount of CSP is expressed on days 7 and 8 *Pf* Oocysts. Thus, if the amount of *Pf* CSP on *Pf* Oocysts can be reliably quantified then alteration in the total amount of CSP expressed on the midgut parasites may serve as an indicator of transmission reducing activity, when the amount of CSP lies within the linear range of detection. This would require knowledge of the amount of *Pf* CSP expressed on *Pf* Oocysts from samples collected at uniform intervals after an infected blood meal.. We applied the ECL-SB to quantitate the amount of *Pf* CSP on day 8 *Pf* Oocysts. This was accomplished in a total of 18 unstained and 18 mercurochrome-stained (microscopy counted) midgut samples. Both stained and unstained midguts were harvested from the same group of mosquitoes that had received a *P. falciparum* infected blood meal.

In order to compare the amount of *Pf* CSP in stained and unstained *Pf* Oocyst samples, we fitted the IOD values obtained from r*Pf*CSP (3.125, 6.25 and 12.5 pg) to the M-M equation (1) and thus acquired numerical values of V_m_ and K. Next, the inverse function of the M-M equation was applied to IOD*_Observed_* values obtained from the equivalent of two *Pf* Oocyst*s.* The inverse function of M-M equation (1) is defined as
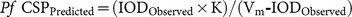
where K>0 and V_m_>IOD_Observed._ This procedure was applied to each of the 18 stained and unstained *Pf* Oocyst samples and the median and the 1^st^ and 3^rd^ quartiles of *Pf* CSP concentration on *Pf* Oocysts were calculated. The Mann-Whitney test was used to detect the difference in the amount of *Pf* CSP estimated in the two samples.

**Figure 6:**
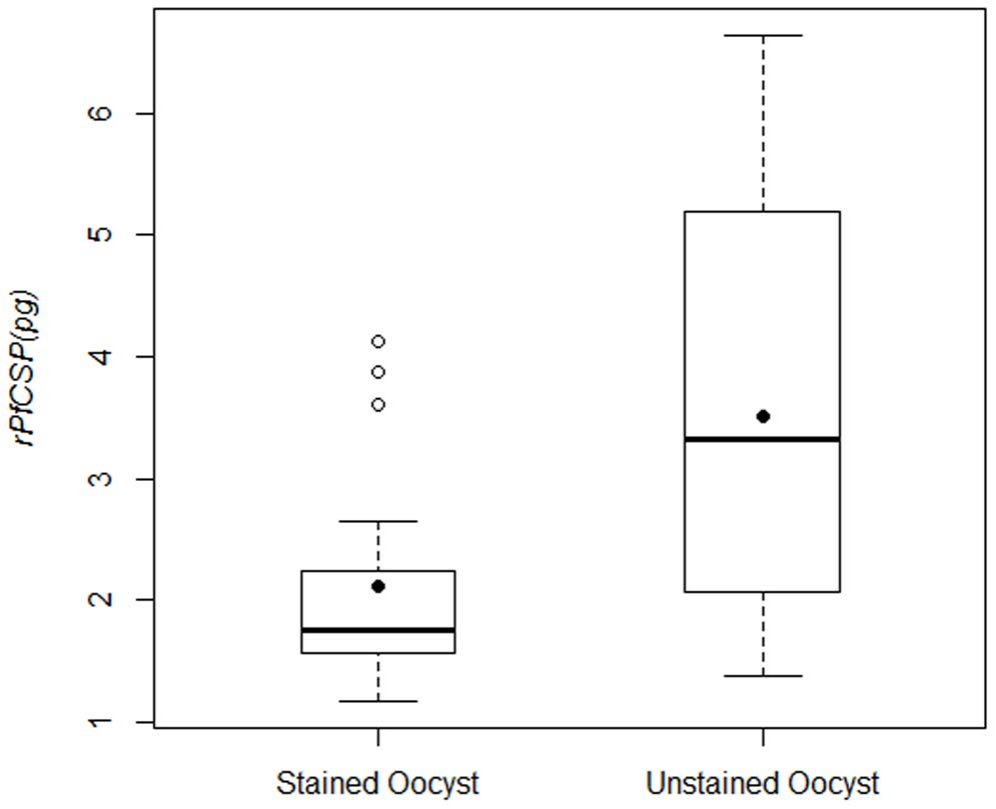
Quantitation of *Pf*CSP expression on *Pf* Oocysts. The expression of *Pf* CSP on day 8 oocysts was estimated in a total of 18 unstained and 18 stained (microscopy counted) midgut samples by ECL-SB and expressed in pg amount. In each assay, samples containing the equivalent of 2 *Pf* Oocysts were used. The filed dot indicates the means of estimated amount of r*Pf* CSP, the bold line represents the median r*Pf* CSP value and the open dots are the outlier values in 3 *Pf* Oocysts.

Based on our calculations, the median and the range between 1^st^ and 3^rd^ quartiles of *Pf* CSP on 2 *Pf* Oocysts were 3.32 pg (2.11 pg, 5.09 pg) for unstained samples and 1.76 pg (1.59 pg, 2.22 pg) for stained samples, respectively (Fig. 6). There is a larger sample-to-sample variation in the unstained *Pf* Oocyst group (1.58–6.63 pg) compared to the stained group (1.18–4.12 pg) that could be attributed to the fact that the counts in the unstained group are assigned values whereas in the stained group they are actual counts determined by microscopy suggesting that pre-stained oocyst samples will be useful for future slot blot assay validation. In addition, the Mann-Whitney test showed a highly significant difference (p-value = 0.00787, 2-sided) in the amount of *Pf* CSP in the unstained and stained groups. Several reasons can be attributed to these differences between the two groups that included more accurate *Pf* Oocyst counts but lower quantifiable CSP in the pre-stained group because mercurochrome staining may have partly interfered with the CSP NANP-epitope reactivity with the mAb 2A10. Nonetheless, based on ECL-SB, a single day 8 *Pf* Oocyst on average produced approximately 2 pg (0.5-3.0 pg) of *Pf* CSP. It is widely recognized that the oocyst intensity (number of oocysts per midgut), prevalence (percent of infected midguts) and their sizes in control groups varies greatly in laboratory settings [9]. Thus, it is reasonable to assume that the amount of *Pf*CSP in *Pf* Oocysts that are in similar developmental stages would also vary inherently. The application of ECL-SB to measure the transmission reducing activity of antibodies generated by natural infection or by vaccination is under investigation.

### Assay Precision

Precision is defined as the repeatability of an assay. For this purpose, we measured the inter-assay and intra-assay variability of r*Pf* CSP and *Pf* CSP on day 8 *Pf* Oocyst in ECL-SB. We used 5 pg of r*Pf*CSP and 4 *Pf* Oocysts for the precision studies. The assay precision was calculated as coefficient of variation (CV).

**Figure 7:**
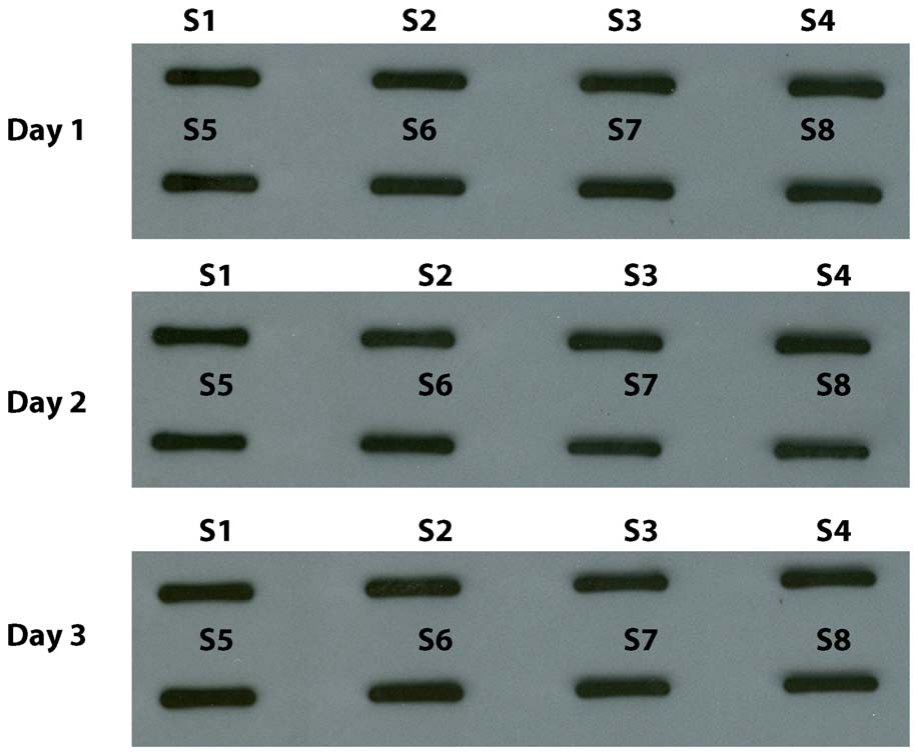
r*Pf* CSP detection - Intra-assay and inter-assay variability. 5pg of r*Pf* CSP in 20 µl were loaded in eight wells using a slot blot apparatus on to nitrocellulose membrane and probed with 2A10 mAb. The r*Pf* CSP and 2A10 mAb reactivity were determined by measuring the intensity of protein band using ImageJ software and were represented as IODs. An experiment with identical design was repeated on three different days and the IOD values were used to calculate intra-assay and inter-assay variability.

The CV (%) for inter-assay variability for r*Pf* CSP detection by ECL-SB was 1.74% (Fig. 7 and Table 1) and 1.32% for *Pf* CSP expression on *Pf* Oocyst (Table 2) which was calculated by using the means of eight replicates obtained on three different days. The CVs (%) for intra-assay variability for r*Pf* CSP detection on three days done on eight replicates were 2.41%, 0.82% and 2% (Table 3) and for *Pf* CSP expression on *Pf* Oocysts on three days run eight times were 1.52%, 0.57%, and 1.86% (Table 4). These results clearly indicate that the ECL-SB is highly precise in detecting *Pf* CSP in recombinant form and as native protein on *Pf* Oocyst.

**Table 1:** r*Pf CSP* detection - Inter assay Variability.

	*IOD x 1000								
Days	S1	S2	S3	S4	S5	S6	S7	S8	Mean
1	2075K	2038K	1992K	2062K	2026K	1951K	2074K	1964K	2023K
2	2077K	2075K	2058K	2052K	2071K	2107K	2071K	2088K	2075K
3	2117K	2180K	2096K	2149K	2052K	2079K	2101K	2072K	2106K
Grand Mean									2068K
SD of Means									36K
** **CV (%)**									**1.7430%**

* Integrated optical density was measured for 5 pg of r*Pf CSP*.

** CV (%)  =  (SD of Means/Grand Mean) x 100.

**Table 2:** *Pf* CSP expression on *Pf* Oocyst detection - Inter assay Variability.

	*IOD x 1000								
Days	S1	S2	S3	S4	S5	S6	S7	S8	Mean
1	2435K	2434K	2416K	2418K	2381K	2343K	2349K	2413K	2399K
2	2426K	2421K	2407K	2384K	2413K	2405K	2408K	2395K	2407K
3	2543K	2482K	2472K	2411K	2412K	2418K	2432K	2438K	2451K
Grand Mean									2419K
SD of Means									32K
** **CV (%)**									**1.3173%**

* Integrated optical density was measured for 4 *Pf* Oocyst*s*.

** CV (%)  =  (SD of Means/Grand Mean) x 100.

**Table 3:** r*Pf* CSP detection - Intra-Assay variability.

	*IOD x 1000		
Samples	Days		
	1	2	3
S1	2075K	2077K	2117K
S2	2038K	2075K	2180K
S3	1992K	2058K	2096K
S4	2062K	2052K	2149K
S5	2026K	2071K	2052K
S6	1951K	2107K	2079K
S7	2074K	2071K	2101K
S8	1964K	2088K	2072K
Mean	2023K	2075K	2106K
SD	49K	17K	42K
** **CV (%)**	**2.4142%**	**0.8241%**	**2.0035%**

* Integrated optical density was measured for 5 pg of r*Pf CSP*.

** CV (%)  =  (SD of Sample IODs/Mean) x 100.

**Table 4:** *Pf* CSP expression on *Pf* Oocyst detection - Intra-Assay variability.

	*IOD x 1000		
Samples	Days		
	1	2	3
S1	2435K	2426K	2543K
S2	2434K	2421K	2482K
S3	2416K	2407K	2472K
S4	2418K	2384K	2411K
S5	2381K	2413K	2412K
S6	2343K	2405K	2418K
S7	2349K	2408K	2432K
S8	2413K	2395K	2438K
Mean	2399K	2407K	2451K
SD	36K	14K	46K
** **CV (%)**	**1.5178%**	**0.5635%**	**1.8616%**

* Integrated optical density was measured for 4 *Pf* Oocyst*s*.

** CV (%)  =  (SD of Sample IODs/Mean) x 100.

A major advantage of ECL-SB is its high level of sensitivity (as little as 1.25 pg for r*Pf*CSP and average of 2 pg on day 8 *Pf* Oocyst). In the SMFA, while infection intensity of>30 oocysts in the control group is achievable, Rosenberg has reported that oocyst counts in wild caught *Anopheles* mosquitoes are generally very low (<5 per mosquito) in areas of moderate to high endemicity [25]. Thus, this assay may be of particular advantage in detecting malaria prevalence in mosquitoes in field settings where the oocyst counts are considerably lower than those observed in the SMFA. A caveat of the CSP-based ECL-SB would be the inability to detect malaria infection in mosquitoes that had received an infectious blood meal prior to day 7 of testing. To increase the window of detection, novel antigens expressed in the midgut oocyst prior to day 7 are being evaluated for their application in ECL-SB. This assay is also being optimized for high throughput adaptation and the use of whole mosquito to obviate the need for dissection for midgut isolation.

### Estimation of *P. falciparum* Prevalence in Mosquito Populations

**Figure 8:**
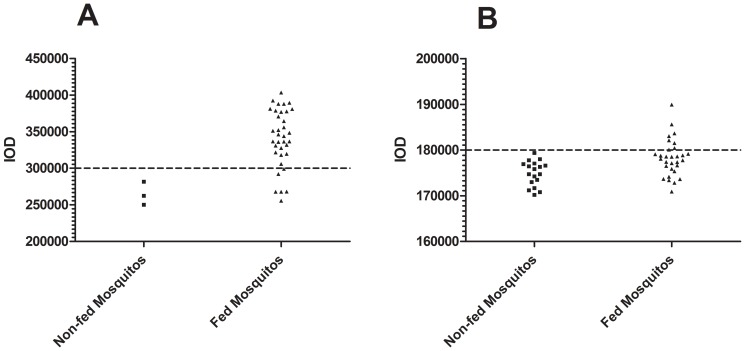
Slot blot estimation of *P. falciparum* prevalence in *Anopheles stephensi* vector populations. A: Individual mosquitos from a population with an estimated prevalence of 85.7% and average oocyst burden of 9.4 oocysts per mosquito were subjected to slot blot analysis. A diagnostic cutoff intensity value was obtained by calculating the mean plus 2 standard deviations of the negative control sample. Employing this criterion, the slot blot assay estimates a prevalence of 83.3% (30/36). B: This analysis was repeated for a mosquito population with a lower oocyst intensity of approximately 0.6 oocysts per mosquito and a prevalence of 27.6%. For these lower prevalence mosquitos, the slot blot estimates a population prevalence of 23.3% (7/30). Non-fed mosquitos: *A. stephensi* mosquitos that did not receive a blood meal spiked with *P. falciparum* gametocytes. Fed mosquitos: *A. stephensi* mosquitos that have received a blood meal spiked with *P. falciparum* gametocytes.

We wanted to know the value of ECL-SB in determining *P. falciparum* prevalence in *Anopheline* mosquitoes. Since the parasite burden in mosquito midguts is known to vary greatly in both laboratory and field settings, we tested the ability of the ECL-SB to estimate overall parasite prevalence in two independent mosquito populations fed either a high (0.035%) or low (0.009%) dilution of *P. falciparum* gametocytes produced in cultures. Microscopic dissection and mercurochrome staining of either 56 or 58 randomly sampled mosquitos from these two different cohorts taken 8 days post blood meal identified average estimated oocyst intensities of 9.4 and 0.6 oocysts per mosquito, respectively. Microscopic counting of stained mosquito samples also established a reference prevalence of 85.7% (48/56) in the high burden (9.4 oocyst per mosquito) and 27.6% (16/58) in the low burden (0.6 oocysts per mosquito) group. Without prior knowledge of these estimates, we employed the ECL-SB and a diagnostic threshold calculated from the mean signal intensities of the unfed control mosquitoes plus two standard deviations to provide a comparative estimate of *P. falciparum* prevalence. The ECL-SB procedure identified 30/36 positive mosquitos from the 9.4 oocyst/mosquito group or a prevalence of 83.3% (Fig. 8A). A chi squared test performed upon these data assuming a null hypothesis of no significant difference between methods yields a p value of 0.756, strongly suggesting that results from microscopy and slot blot analysis represent the same overall distribution. The IOD values in the lower prevalence 0.6 oocyst/mosquito population were closer to the those obtained in the unfed mosquito population (Fig. 8B), however a number of infected mosquitos were still clearly distinguishable by ECL-SB; 7/30 mosquitos in this group were classified as positive for the given threshold resulting in a prevalence of 23.3% (Fig. 8B). Chi squared analyses of these diagnostic classifications leads to a p value of 0.80, again reiterating the strong correlation between the results of microscopy and ECL-SB. Cumulatively, the estimates of parasite prevalence acquired via slot blot analysis were very similar to those estimated via direct midgut dissection and microscopic counting method. These data thus demonstrate the ECL-SB may be an effective tool for determining the prevalence of *P. falciparum* infection in mosquito populations. Future applications of this assay may include measuring the impact of vaccines, drugs or other interventions on reduction in malaria infectivity in mosquitoes in laboratory and field settings. Established benefits of the current assay are high sensitivity, reproducibility, ease of operation, and potential for high throughput adaptation. In addition, due to the invariant nature of the *Pf*CSP-NANP epitope, we expect this ECL-SB to be suitable for detection of *Pf*CSP across *P. falciparum* strains and in susceptible *Anopheles* vectors, and experiments are underway to confirm this. One possible confounder could be the reported variations in the copy number of NANP repeats on *Pf*CSP in the field isolates. In a recent study, 25-49 copies of *Pf*CSP NANP repeats were reported in Malawi [26]. Whether these variant copy numbers of NANP repeats would influence the performance of an assay based on measuring the level of PfCSP expression needs to be determined.
